# Adaptive Evolution of the *FADS* Gene Cluster within Africa

**DOI:** 10.1371/journal.pone.0044926

**Published:** 2012-09-19

**Authors:** Rasika A. Mathias, Wenqing Fu, Joshua M. Akey, Hannah C. Ainsworth, Dara G. Torgerson, Ingo Ruczinski, Susan Sergeant, Kathleen C. Barnes, Floyd H. Chilton

**Affiliations:** 1 Division of General Internal Medicine, Department of Medicine, The Johns Hopkins University, Baltimore, Maryland, United States of America; 2 Division of Allergy and Clinical Immunology, Department of Medicine, The Johns Hopkins University, Baltimore, Maryland, United States of America; 3 Department of Genome Sciences, School of Medicine, University of Washington, Seattle, Washington, United States of America; 4 Department of Biochemistry, Wake Forest University Health Sciences, Winston-Salem, North Carolina, United States of America; 5 Wake Forest Center for Botanical Lipids and Inflammatory Disease Prevention, Wake Forest University Health Sciences, Winston-Salem, North Carolina, United States of America; 6 Department of Medicine, University of California San Francisco, San Francisco, California, United States of America; 7 Department of Biostatistics, Bloomberg School of Public Health, Johns Hopkins University, Baltimore, Maryland, United States of America; 8 Department of Physiology/Pharmacology, Wake Forest University Health Sciences, Winston-Salem, North Carolina, United States of America; Rikagaku Kenkyūsho Brain Science Institute, Japan

## Abstract

Long chain polyunsaturated fatty acids (LC-PUFAs) are essential for brain structure, development, and function, and adequate dietary quantities of LC-PUFAs are thought to have been necessary for both brain expansion and the increase in brain complexity observed during modern human evolution. Previous studies conducted in largely European populations suggest that humans have limited capacity to synthesize brain LC-PUFAs such as docosahexaenoic acid (DHA) from plant-based medium chain (MC) PUFAs due to limited desaturase activity. Population-based differences in LC-PUFA levels and their product-to-substrate ratios can, in part, be explained by polymorphisms in the fatty acid desaturase (*FADS*) gene cluster, which have been associated with increased conversion of MC-PUFAs to LC-PUFAs. Here, we show evidence that these high efficiency converter alleles in the *FADS* gene cluster were likely driven to near fixation in African populations by positive selection ∼85 kya. We hypothesize that selection at *FADS* variants, which increase LC-PUFA synthesis from plant-based MC-PUFAs, played an important role in allowing African populations obligatorily tethered to marine sources for LC-PUFAs in isolated geographic regions, to rapidly expand throughout the African continent 60–80 kya.

## Introduction

Studies suggest that anatomically modern humans arose in Africa approximately 150 thousand years ago (kya), expanded throughout Africa ∼60–80 kya, and to most parts of Europe and Asia ∼40 kya[1]–[6]. Numerous mitochondrial DNA studies support what Foster and Matsumera [5] describe as a ‘remarkable expansion’ from a small geographic region dating broadly to ∼60–80 kya. Interestingly, this expansion occurred at a period of time when archeological evidence indicates great advances in technological, social, and cognitive behavior [7]. Pivotal genetic and/or environmental (which led to shifts in adaptive and selective pressures) changes that enabled the dramatic expansions first within Africa and then throughout the world remain unknown.

The brain is ∼60% (dry weight) lipid and is highly enriched in relatively rare (in nature) LC-PUFAs critical for proper brain structure and function, especially docosahexaenoic acid (DHA) and arachidonic acid (AA). Long chain polyunsaturated fatty acids (LC-PUFAs) are essential for brain structure, development, and function, and adequate dietary quantities of LC-PUFAs are thought to have been necessary for both brain expansion and the increase in brain complexity observed during modern human evolution [8]. In fact, LC-PUFAs have long been considered to be the most limiting nutrients for neural growth and complexity during fetal and early childhood development, but they are not widely available in foods [9], [10]. Additionally, metabolic studies to date indicate that humans have little capacity to synthesize LC-PUFAs, especially DHA, from plant-based MC-PUFAs [11], [12]. Consequently, an unresolved question is how enough DHA and AA were acquired to support large brains and increases in brain complexity throughout human evolution[13]–[15]. The prevailing view is that early human African populations obtained sufficient concentrations of LC-PUFAs to support brain evolution from DHA-enriched marine sources by living at the margins of lakes, rivers, or seashores in central and eastern Africa [8]. But what facilitated the movement away from stable sources of DHA and expansions into a wide range of environmental (including arid) conditions?

**Figure 1:**
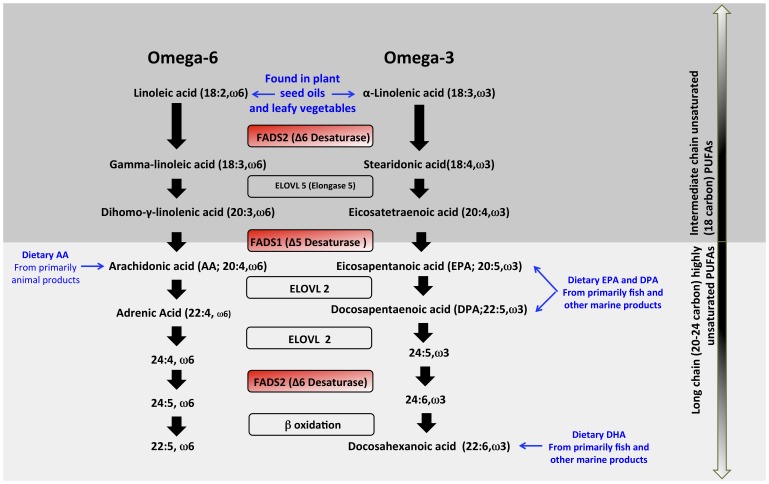
Overview of PUFA metabolism illustrating the critical involvement of *FADS1* and *FADS2* genes in desaturation steps necessary for the metabolism of intermediate chain unsaturated PUFAs (top dark gray panel) to long chain highly unsaturated PUFAs (bottom light gray panel). Omega 3 (right) and Omega 6 (left) pathways are illustrated along with known genes (in center rectangles) and dietary sources of the PUFAs.

We recently reported that human populations differ dramatically in their capacities to synthesize LC-PUFAs from plant-based MC-PUFAs. Specifically, we found higher levels of LC-PUFAs in African-American individuals compared to European-Americans, which is attributable in part to variation in the *FADS* gene cluster on chromosome 11q12–13 [16], [17]. These enzymes are responsible for three desaturation steps in DHA synthesis (**Figure 1**) and have long been recognized as rate-limiting in the conversion of MC-PUFAs to LC-PUFAs (reviewed in [18]). To date, however, the evolutionary history of functionally important variation in the *FADS* gene cluster has not been characterized, particularly in African populations and we investigate this in this work.

## Results

**Figure 2:**
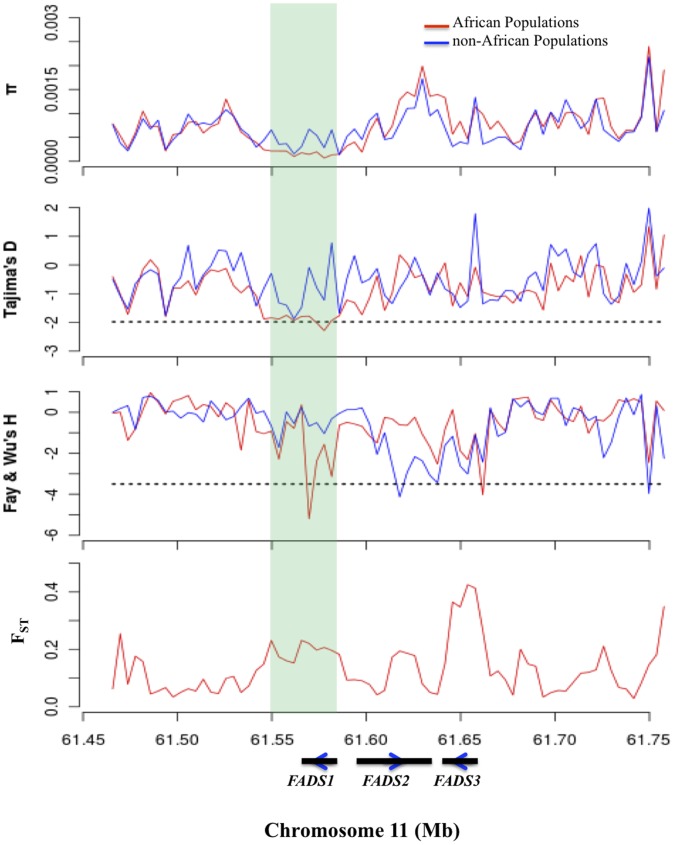
Summary of sliding window analysis across a 300 kb region (chr11∶61467097–61759006, hg19) centered on the *FADS* gene cluster for two African (YRI and LWK) versus eight non-African populations (IBS, CEU, GBR, FIN, TSI, JPT, CHB and CHS). Genetic diversity π, Tajima’s D, Fay & Wu’s H, and pairwise F_ST_ were calculated using a window size of 5 kb and an overlap of 1 kb. The teal shaded box represents the ∼30 kb haplotype block noted within the African samples and the three black bars represent *FADS1, FADS2* and *FADS3* from left to right, respectively along with direction of transcription. Dotted lines represent the threshold for an empirical P = 0.01 comparing across all windows in the genome for Tajima’s D, Fay & Wu’s H.

To investigate the evolutionary forces shaping patterns of variation in the *FADS* gene cluster in geographically diverse populations, we analyzed 1092 individuals representing 14 populations sequenced as part of the 1000 Genomes Project (1 KGP; population-specific details shown in **Table S1**) and focused on a 300 kb region centered on the *FADS* loci (chr11∶61467097–61759006, hg19). To evaluate whether patterns of genetic variation at the FADS loci are consistent with natural selection, we calculated Tajima’s D and Fay and Wu’s H in 5 kb sliding windows across the genome. We obtained empirical p-values by comparing Tajima’s D and Fay and Wu’s H at the *FADS* loci to the remainder of the genome to look for deviations in these statistics beyond that explained by demographic history. **Figure 2** summarizes the distribution of these statistics in African versus non-African populations in the 300 kb region of interest relative to the rest of the genome; details on all the populations are shown in **Figure S1**. A pattern consistent with positive selection was observed in African populations (**Figure 2**), with the strongest signal within *FADS1* (Tajima’s D  =  −2.38, empirical *P* = 0.0006; Fay & Wu’s H  =  −5.20, empirical *P* = 0.0011). Furthermore, levels of nucleotide diversity, measured by π, were decreased and large allele frequency differences between African and non-African populations exist in the *FADS1* region, hallmarks of a classic selective sweep. Within the African admixed ASW population (**Figure S1**), we noted similar signatures of selection, albeit not significant, possibly due to European admixture. Haplotype structure in this chromosomal region in the African populations reveals a haplotype block (using the confidence intervals approach) with high LD of ∼30 kb (chr11∶61551356–61581764, hg19), which includes *FADS1* (**Figure 2**).

**Figure 3:**
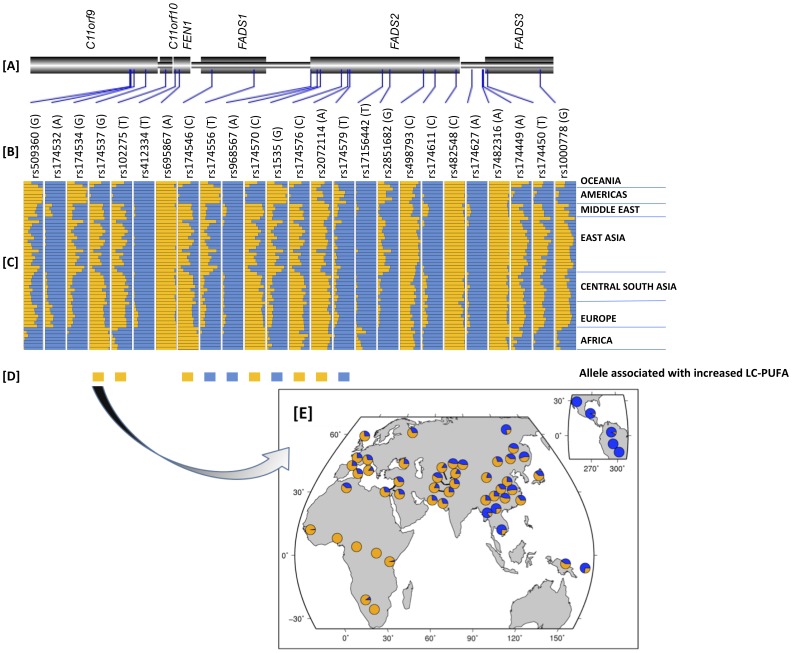
Geographic distribution of *derived* allele frequencies in a 100 **kb region surrounding rs174537 in the 52 populations represented in the Human Genome Diversity Panel Data.** **Panel A** represents physical position of the SNPs relative to genes in the region, **Panel B** is SNP name (derived allele), **Panel C** is frequency of derived allele (in orange) in the populations clustered based on geography, **Panel D** is an indication of the allele associated with increased LC-PUFA metabolism in published association studies, and Panel E is the detailed overview of rs174537 showing is near fixation within Africa.

**Figure 4:**
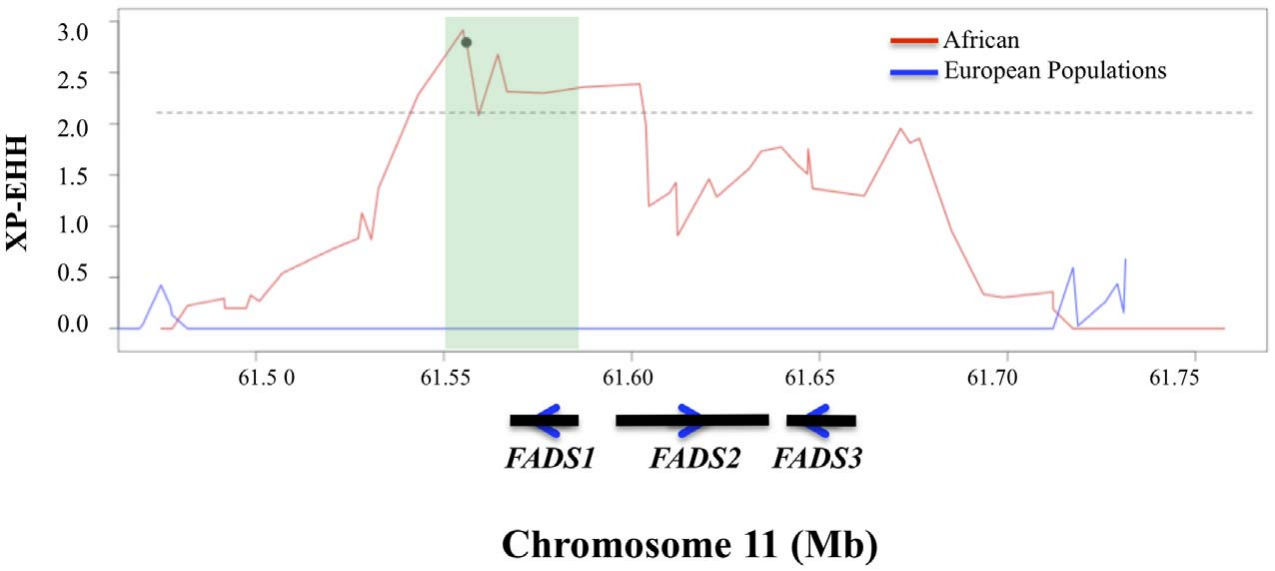
XP-EHH scores across the 300kb region (chr11∶61467097–61759006, hg19) around the *FADS* gene cluster in populations from Africa (blue) and Europe (red) within the HGDP. SNP rs174737 is illustrated with the black dot on the African curve. The teal shaded box represents the ∼30 kb haplotype block noted within the African samples and the three black bars represent *FADS1, FADS2* and *FADS3* from left to right, respectively along with direction of transcription. Dotted line represents the threshold for an empirical P = 0.01 comparing across all windows in the genome for XP-EHH.

To provide additional support for the hypothesis of positive selection acting on the *FADS1* region in African populations, we next assessed patterns of LD and haplotype structure in the Human Genome Diversity Panel (HGDP) [19]. We found that alleles (**Figure 3**) which are associated with *enhanced* PUFA metabolism [17], [18] for most of the SNPs in a 100 kb region encompassing the *FADS* loci are at higher frequency within Africa as compared to other populations. Strikingly, the *derived* allele (G) at rs174537, selected for illustration simply as it is the SNP that exhibits the peak association signal for LC-PUFA metabolism [18], [20], emblematic of other variants within the haplotype block noted above, is fixed within Africa, but is at intermediate frequencies in non-African populations (**Figure 3**). Consistent with signatures of selection in the 1 KGP data, XP-EHH scores (a measure of differences in LD between populations) from the HGDP in the same 300 kb region described above (**Figure 4**) are also highly supportive of recent positive selection within Africa (XP-EHH = 2.91, empirical P = 0.0008 derived by comparision to all other windows across the genome), with no evidence of selection outside Africa. A selective sweep at or near rs174537 within the African continent is likely complete or nearly complete, as we find little evidence for selection within Africa based on the integrated Haplotype Score (iHS, data not shown), and because the derived allele appears to have almost gone to fixation.

**Figure 5:**
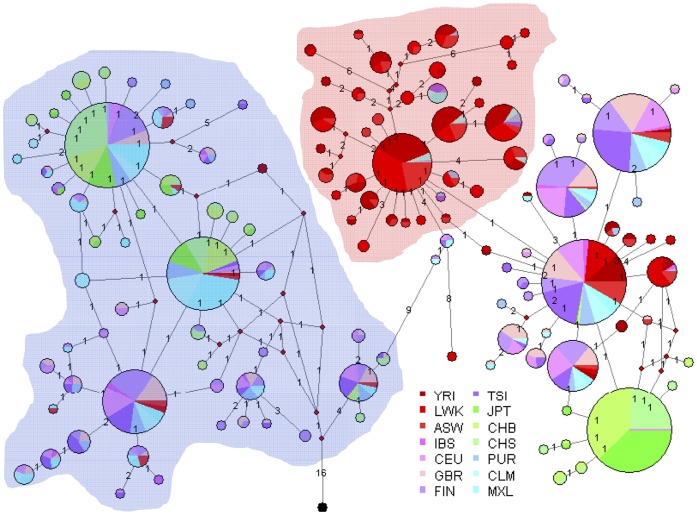
Median-joining network for the relationship of haplotypes of 1,092 individuals in a ∼30 **kb block of LD including** ***FADS1*** **.** Circles represent haplotypes with an area proportional to frequency. Singleton haplotypes were not shown. “Ancestral” is a reconstructed haplotype carrying the ancestral (chimpanzee) allele at each position as illustrated in black.

Peak phenotype association [17] observed in African-Americans includes rs174537 and much of *FADS1*; rs174537 has the strongest p-value with LC-PUFAs reported to date in the published literature [18]. Importantly, the ∼30 kb haplotype block, detected in the 1 KGP above, includes *FADS1* (teal blocks in **Figures 2** **and** **4**), coincides with the peak positive selection signal, overlaps completely with the peak association signal with LC-PUFA noted in African Americans [17], includes rs174537, and includes three known eQTLs for *FADS1* [21] (rs174547, rs174548 and rs174549; rs174548 is also a reported eQTL for *FADS2* and *FADS3*). A Median-joining network (**Figure 5**) of the haplotypes within this block show that only 13.6%, 20.1% and 22.1% of haplotypes in YRI, LWK, and ASW, respectively, are in the ancient haplotype group. The mean number of mutations from the “ancestral” (i.e., chimpanzee) to the 2184 human haplotypes was estimated to be 23.78 (SEM = 3.73). Given 207 fixed differences between chimpanzee and human in this region, we estimate a TMRCA of 1.49 (SEM = 0.23) million years for the human haplotypes. Similarly, only considering the number of mutations within the haplotype group D1, the TMRCA was 85,000±84,000 years, thus suggesting that selection in Africa occurred approximately 85 kya.

## Discussion

The current work confirms marked global differences in the allele frequencies of variants in the *FADS* gene cluster that was first noted in African Americans and European Americans [16], [17], especially at variants strongly associated with the *efficiency* of conversion of LA and ALA to AA and DHA, respectively. Two independent samples of global genetic variation, the 1KGP and HGDP, reveal empiric evidence for signatures of positive selection above the 99^th^ percentile of the genome in the 300 kb region centered on the *FADS* loci, making them strong candidates for having been subject to recent positive selection [19]. Jointly, these two sets of data support the hypothesis that advantageous mutations within the *FADS* gene cluster occurred prior to human migration out of Africa (∼85 kya), and swept to fixation within African but not European or Asian populations. Furthermore, multiple studies prove unequivocally that the *derived* alleles are associated with *enhanced* metabolism of MC-PUFA to LC-PUFAs, suggesting this is the driving force behind positive selection at the *FADS* gene cluster.

Archeological evidence for regular active hunting of large animals emerged about 50 thousand years ago (kya); by 12–14 kya, humans begin using fishhooks, bows and arrows; and by 10 kya, they began to domesticate plants and animals [22]. Each of these advances would have ensured that humans had much more reliable dietary sources of LC-PUFAs (e.g., meat, eggs and fish). As to why the evidence of selective pressure and a selective sweep is restricted to within Africa and not beyond, we can speculate that perhaps as small groups of humans carrying the ancestral allele migrated out of Africa ∼40–50 kya, the selective pressure to make LC-PUFA was diminishing as the social and technological capacity to obtain them from their environment markedly increased. Consequently, it is likely that at some point, LC-PUFA synthesis may have become too metabolically expensive when it was readily available in common food sources thereby leading to the maintenance of the ancestral alleles in the non-African populations included here in our study.

In summary, our results provide support for the hypothesis that positive selection acted to sweep derived alleles in the *FADS* region, which are associated with *enhanced* metabolism of MC-PUFA to LC-PUFAs, to near fixation in African populations. There has been considerable debate on how early humans escaped the developmental vulnerability to obtain sufficient DHA and AA necessary to maintain brain [8], [13], [23], [24] size and complexity, especially in light of studies suggesting that only trace amounts of LC-PUFAs could have been synthesized from plant-derived sources [25]. The evidence presented here from the 1 KGP and HGDP data suggest that a ‘game changing’ event (one or more mutations in the *FADS* cluster), likely occurred early at a time that could have dramatically impacted the rapid expansion from central source populations, ∼60–80 kya. Klein has suggested that modern patterns of culture and technology were due to a sudden change in cognitive capacities entailing some form of neurological mutation [26] which Mellars suggests occurred ∼80 kya [7]. While it is not possible to determine the cognitive impact of a mutation in the *FADS* cluster ∼84 kya, it is likely that suddenly having the capacity to more efficiently convert plant-based MC-PUFA to LC-PUFAs would have been an important advantage that would have facilitated expansion and movement into a variety of ecological locations.

## Materials and Methods

### Analysis of the 1KGP Data

To fine map the signature of positive selection of the *FADS* gene cluster in Africans, resequencing data in a 300 kb around this region (chr11∶61467097–61759006, hg19) were analyzed from the Thousand Genomes Project (1 KGP, Phase1integrated variant call set, http://www.1000genomes.org/). A total of 1,092 individuals from 14 populations were included (**Table S1**). We calculated genetic diversity (π), test statistics from site-frequency-spectrum based neutral tests (i.e., Tajima’s D and Fay & Wu’s H) [27], [28], and population differentiation (pairwise F_ST_ [29]) using 5 kb sliding windows with an overlap of 1 kb. Positive selection can result in a deficit of genetic diversity and an excess of rare variants as measured by Tajima’s D. However, background selection can have a similar effect on genetic diversity as a selective sweep due to positive selection, making it difficult to distinguish between the two [30]. Therefore, we also compared measures of Fay & Wu’s H to better indicate an excess of high-frequency derived variants to gain a more comprehensive view of positive selection, and to distinguish between a selective sweep versus background selection. In order to assess the significance for each 5 kb window within the 300 kb region of interest, for each statistic we used the empirical distribution across all 5 kb windows across the genome to minimize the bias caused by low-coverage sequencing [31]. Phasing information for the resequencing data set was obtained from 1 KGP, and haplotype structure and LD blocks in the region was defined using standard confidence intervals algorithms in Haploview [32]. Only single nucleotide polymorphisms were considered and chimpanzee was treated as an outgroup. Median-joining network was constructed for the selected haplotype block using Network 4.61 (http://www.fluxus-engineering.com/sharenet.htm) [33]. Time to the most recent common ancestor (TMRCA) was estimated based on this network [34] assuming a chimpanzee-human split 6.5 million years ago.

### Analysis of the HGDP Data

Genotypes from 1,043 samples from 52 populations in the Human Genome Diversity Panel (HGDP) available from the HGDP genome browser (http://hgdp.uchicago.edu/) [19] were used to evaluate evidence for positive selection around the *FADS* gene cluster (chr11∶61467097–61759006, hg19). Two haplotype-based tests were used to evaluate the degree of evidence for recent positive selection in the HGDP: the integrated Haplotype Score (iHS) [35], and the Cross Population Extended Haplotype Homozygosity (XP-EHH) [36]. The iHS is useful for identifying partial selective sweeps (or sweeps in progress) by identifying advantageous alleles at common SNPs that reside on unusually long haplotypes due to the actions of positive selection. However, the iHS has reduced power to detect selection as the advantageous allele approaches fixation. Therefore, we also examined the XP-EHH statistic that includes a comparison to a reference population, making it more powerful for identifying completed or almost completed selective sweeps whereby the advantageous allele is almost fixed in one population but polymorphic in the human population as a whole. Data on both these statistics and allele frequencies were downloaded from the HGDP selection browser.

## Supporting Information

Figure S1 Summary of sliding window analysis across the 300 kb region (chr11∶61467097–61759006, hg19) region around the *FADS* gene cluster for each individual 1 KGP population. Genetic diversity π (**Panel A**), Tajima’s D (**Panel C**), Fay & Wu’s H (**Panel E**), and pairwise Fst between YRI and other populations (**Panel B**) and that between LWK and other populations (**Panel D**) based upon a window size of 5 kb and an overlap of 1 kb. The horizontal black bar represents *FADS1*.
(TIFF)

Table S1 Sample information for 1,092 unrelated individuals from the 1000 Genomes Project.
(DOCX)
