# Supplementary figures and images for: Adaptive Evolution of the FADS Gene Cluster within Africa
Source: PLoS One. 2012 Sep 19;7(9):e44926. doi: 10.1371/journal.pone.0044926 (PMC3446990; doi:10.1371/journal.pone.0044926)

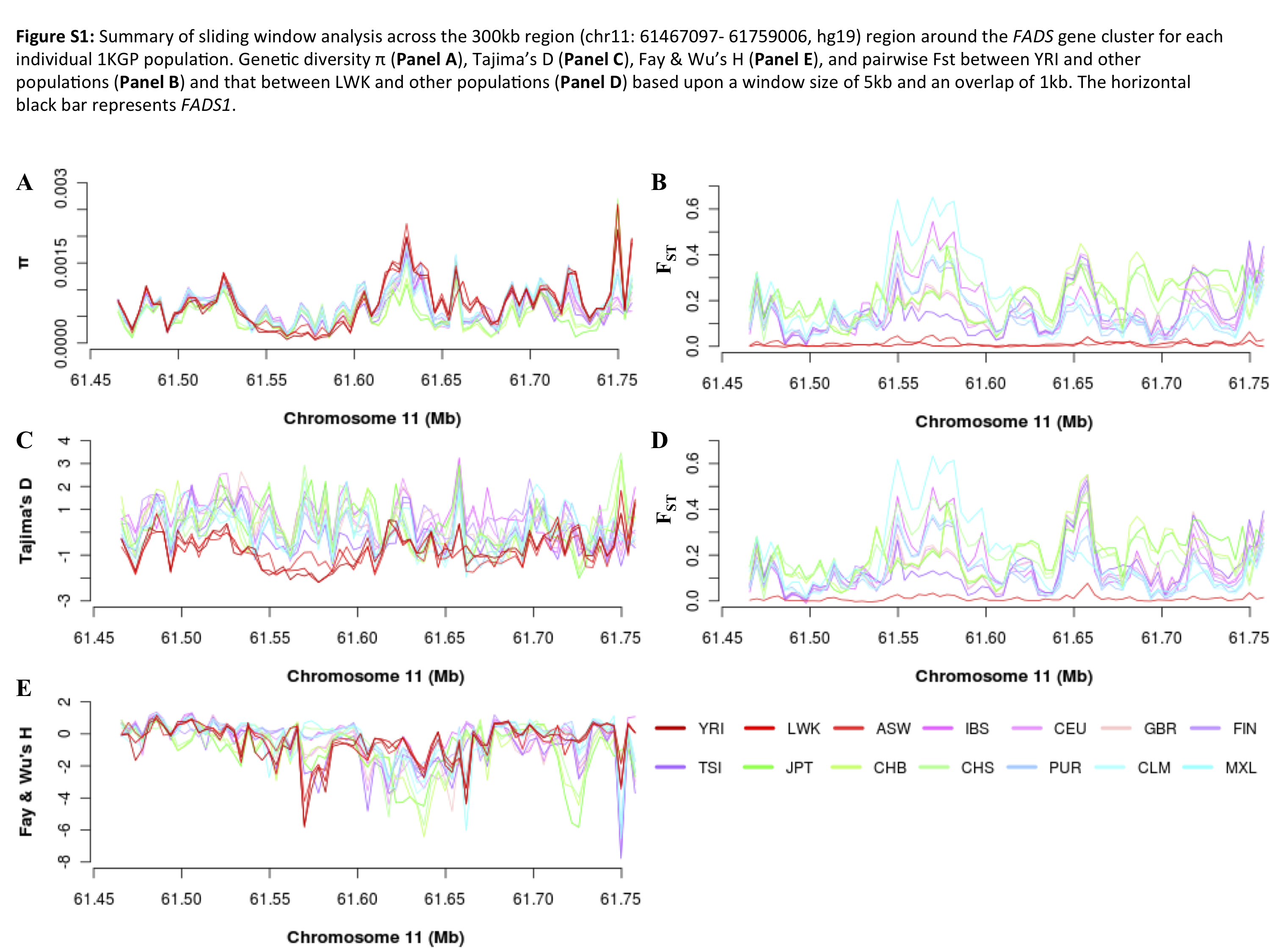

Supplement: Figure S1 — Summary of sliding window analysis across the 300 kb region (chr11∶61467097–61759006, hg19) region around the FADS gene cluster for each individual 1 KGP population. Genetic diversity π (Panel A), Tajima’s D (Panel C), Fay & Wu’s H (Panel E), and pairwise Fst between YRI and other populations (Panel B) and that between LWK and other populations (Panel D) based upon a window size of 5 kb and an overlap of 1 kb. The horizontal black bar represents FADS1. (TIFF) [file pone.0044926.s001.tiff]
