# Supplementary material for: Adaptive Evolution of the FADS Gene Cluster within Africa
Source: PLoS One. 2012 Sep 19;7(9):e44926. doi: 10.1371/journal.pone.0044926 (PMC3446990; doi:10.1371/journal.pone.0044926)
Supplement: Table S1 — Sample information for 1,092 unrelated individuals from the 1000 Genomes Project. (DOCX) [file pone.0044926.s002.docx]

**Table S1.** Sample information for 1,092 unrelated individuals from the 1000 Genomes Project.

| **Ancestry** | **Population** | **Description** | **Sample Size** |
| --- | --- | --- | --- |
| Africa | YRI | Yoruba in Ibadan, Nigeria | 88 |
|  | LWK | Luhya in Webuye, Kenya | 97 |
| Europe | IBS | Iberian populations in Spain | 14 |
|  | CEU | Utah residents with ancestry from northern and western Europe | 85 |
|  | GBR | British from England and Scotland | 89 |
|  | FIN | innish in Finland | 93 |
|  | TSI | Toscani in Italia | 98 |
| East Asia | JPT | Japanese in Tokyo, Japan | 89 |
|  | CHB | Han Chinese in Beijing, China | 97 |
|  | CHS | Han Chinese South, China | 100 |
| Americas | MXL | Mexican Ancestry in LA, CA, USA | 66 |
|  | ASW | African Ancestry in SW USA | 61 |
|  | CLM | Colombian in Medellín, Colombia | 60 |
|  | PUR | Puerto Rican in Puerto Rico | 55 |
